# Research on Autonomous Vehicle Path Planning Algorithm Based on Improved RRT* Algorithm and Artificial Potential Field Method

**DOI:** 10.3390/s24123899

**Published:** 2024-06-16

**Authors:** Xiang Li, Gang Li, Zijian Bian

**Affiliations:** School of Automobile and Traffic Engineering, Liaoning University of Technology, Jinzhou 121001, China; 221285015@stu.lnut.edu.cn (X.L.); bianzijian295@163.com (Z.B.)

**Keywords:** autonomous vehicle, RRT* algorithm, path planning, fusion algorithm, curvature, artificial potential field method

## Abstract

For the RRT* algorithm, there are problems such as greater randomness, longer time consumption, more redundant nodes, and inability to perform local obstacle avoidance when encountering unknown obstacles in the path planning process of autonomous vehicles. And the artificial potential field method (APF) applied to autonomous vehicles is prone to problems such as local optimality, unreachable targets, and inapplicability to global scenarios. A fusion algorithm combining the improved RRT* algorithm and the improved artificial potential field method is proposed. First of all, for the RRT* algorithm, the concept of the artificial potential field and probability sampling optimization strategy are introduced, and the adaptive step size is designed according to the road curvature. The path post-processing of the planned global path is carried out to reduce the redundant nodes of the generated path, enhance the purpose of sampling, solve the problem where oscillation may occur when expanding near the target point, reduce the randomness of RRT* node sampling, and improve the efficiency of path generation. Secondly, for the artificial potential field method, by designing obstacle avoidance constraints, adding a road boundary repulsion potential field, and optimizing the repulsion function and safety ellipse, the problem of unreachable targets can be solved, unnecessary steering in the path can be reduced, and the safety of the planned path can be improved. In the face of U-shaped obstacles, virtual gravity points are generated to solve the local minimum problem and improve the passing performance of the obstacles. Finally, the fusion algorithm, which combines the improved RRT* algorithm and the improved artificial potential field method, is designed. The former first plans the global path, extracts the path node as the temporary target point of the latter, guides the vehicle to drive, and avoids local obstacles through the improved artificial potential field method when encountered with unknown obstacles, and then smooths the path planned by the fusion algorithm, making the path satisfy the vehicle kinematic constraints. The simulation results in the different road scenes show that the method proposed in this paper can quickly plan a smooth path that is more stable, more accurate, and suitable for vehicle driving.

## 1. Introduction

The casualties and economic losses caused by road traffic accidents are increasing year by year. Every year, there are traffic accidents caused by improper driving behavior and other related reasons [1]. Driverless cars can well reduce road congestion and greatly reduce traffic accidents caused by human errors [2].

One of the crucial technologies for self-driving vehicles is path planning [3]. It plans the safe driving path according to the map information, sensor data, and target location information and controls the steering and speed of the vehicle to ensure that the vehicle runs safely and efficiently to the destination [4]. The commonly used path-planning algorithms are the intelligent method, the random sampling method, the curve interpolation method, and so on. It can also be classified by global path planning and local path planning. The global path planning algorithm has two methods: random sampling and graph-based search, such as the Dijkstra algorithm [5], the A* algorithm [6], PRM algorithm [7], and the RRT* algorithm [8]. Among them, the RRT* algorithm finds extensive application in the realm of mobile robots and unmanned vehicles, and many researchers have improved the RRT* algorithm. The RRT* algorithm proposed by Karaman et al. [9] introduces the functions of a reselection parent node and reconnection to optimize the search results. However, its search efficiency is suboptimal. Cong et al. [10] proposed an RRT* algorithm based on a hybrid sampling strategy, which effectively reduced the sampling randomness of the algorithm. The HBAI-RRT* algorithm proposed by Lin et al. [11] in their study combines the greedy heuristic method with an adaptive adjustment strategy to reduce the sampling area and improve search efficiency. Local path planning algorithms more frequently employ the artificial potential field (APF) method, which can lead to local minima and goal unreachability issues during the planning process [12]. After conducting an in-depth study, Li et al. [13] adopted the concept of the artificial potential field and improved strategies such as adding distance adjustment factors to address the limitations of the traditional artificial potential field method. Xu [14] engineered a spring mechanism in the repulsive velocity potential field to effectively eliminate the flutter phenomenon near the obstacle. Zhai [15] and others added an adjustment factor and a judgement coefficient to the potential field to optimize the potential field function and improve driving comfort and the safety of the planned path. Many other researchers have proposed fusion algorithms combining the advantages of the two algorithms. Huang [16] introduced a fusion algorithm that merges the RRT algorithm with the artificial potential field method. The expansion of the RRT random tree incorporates probability values and a gravity component. With this method, simulation results demonstrate notable improvements in time efficiency, path length, and iteration count. Zhang [17] introduced a novel path-planning algorithm that enhances obstacle avoidance speed and quality by combining the A* algorithm with the artificial potential field method. This fusion also effectively addresses the inefficiencies of the A* algorithm when dealing with complex scenes. Wu [18] proposed a path planning algorithm that integrates the artificial potential field method with the particle swarm optimization algorithm. This approach allows for the real-time generation of obstacle avoidance paths and significantly improves the vehicle’s stability when avoiding obstacles. Dasiah et al. [19] proposed an improved RRT* algorithm that realizes directional fast search based on sampling angle constraints, which can effectively find better paths in complex environments and significantly improve the convergence rate of the algorithm. In terms of optimality and optimization in path planning problems, Nguyen et al. [20] proposed a research method that integrates formation control and optimal control, taking into account the kinematic and dynamic models of each vehicle. Experiments showed the effectiveness of the control strategy. Shi et al. [21] studied the distributed time-varying output formation tracking problem of heterogeneous multi-agent systems with different dimensions and parameters. They designed an optimal tracking controller by adopting model-free reinforcement learning technology and designing the compensation input for each follower. The simulation results show that the output formation tracking error eventually approaches zero.

Although the above scholars have improved the path planning algorithm, there are still problems that cannot deal with the unknown obstacles emerging in the environment and cannot be applied to structured roads. Although some scholars consider real-time obstacle avoidance, the vehicle collision range is not taken into account, and the final path is not optimized. After consulting the literature, there is little research on the path planning algorithm in the curved structured road scene, and there are some problems, such as low search efficiency. Compared with the optimization of the path tracking control in study [20] and study [21], this paper mainly studies the efficiency and quality of the planned path of an unmanned vehicle, which provides a high-quality path for tracking control and improves the efficiency and stability of tracking. Therefore, this paper designs a method of vehicle driving guided by global path nodes and local planning to avoid obstacles. In order to solve the above path planning problems, this is necessary. The RRT* global path planning algorithm with more sampling nodes is adopted to ensure that more nodes play a guiding role, and the APF local path planning algorithm, which is better at considering the collision range of obstacles and structured road constraints, is adopted to solve the path planning problem of unknown obstacles on structured curved roads. This paper proposes a fusion algorithm that combines the Improved RRT* algorithm and the Improved Artificial Potential Field method. The objective is to tackle the path planning challenge that arises when encountering a prominently unknown obstacle on a structured road. By introducing the concepts of gravitational and repulsive fields into RRT* node sampling, adding probabilistic sampling optimization, and enhancing the purposefulness of node sampling, we improve the RRT* algorithm. Considering the curvature of the road and designing the adaptive step size, which makes the sampling more efficient and time-consuming and solves the problem of possible oscillation when expanding near the target point, subsequently, the nodes of the planned path undergo branching and constraints to finalize the path post-processing phase, enhancing the overall path quality. Enhancements to the artificial potential field method include the design of obstacles, optimization of obstacle avoidance constraints, addition of a road boundary repulsive potential field and optimization of the repulsive function, and the introduction of virtual gravitational points. The goal unreachability problem is solved, and the local optimum is reduced to improve the quality of locally planned paths. Finally, the fusion algorithm significantly improves the performance of obstacle avoidance on structured roads. The planned roads are smoothed to ensure that they are suitable for vehicles.

## 2. Traditional Algorithm

### 2.1. RRT* Algorithm

The RRT algorithm conducts random sampling in unoccupied space and continuously expands random tree branches until a branch contains or approaches the target point, or until the algorithm reaches its iteration limit, at which point the planning process concludes [22].

From reference [9], it is known that the RRT* algorithm has the following theorems:

**Theorem 1.**  (asymptotic optimality of the RRT* algorithm): when the number of iterations tends to infinity, the path quality of the RRT* algorithm tends to be optimal; that is, the algorithm has asymptotic optimality.

**Theorem 2.**  (probabilistic completeness of the RRT* algorithm): if there is a feasible path from the starting point to the end point, the RRT algorithm will almost certainly find a feasible path with an infinite number of iterations.

**Figure 1:**
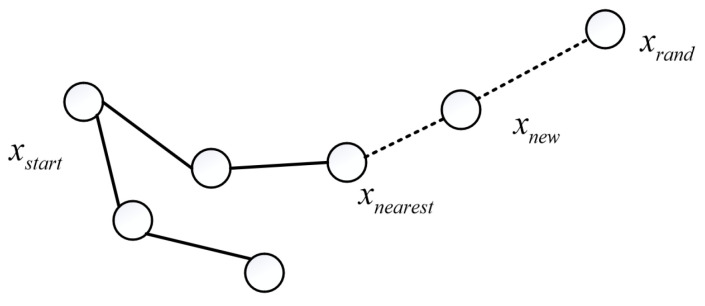
The node extension process.

In the RRT algorithm, the root node of the random tree is the starting point $x_{start}$, and a random point $x_{rand}$ is generated in the blank area of the map. Then, the whole random tree is searched, and $x_{nearest}$ is selected as the nearest node to $x_{rand}$. Connect $x_{rand}$ and $x_{nearest}$, and expand a new node on the connected straight line at a set step size of $x_{new}$. Next, we subject the new node to collision detection. If it does not collide with an obstacle, we add it to the randomly expanded tree. If a collision occurs, then we delete the new node. Continue the described steps until either the target node is appended to the random tree node or the search concludes upon reaching the specified number of iterations. The planned path is derived by retracing from the target point to the starting point. The random sampling process is illustrated in Figure 1.

**Figure 2:**
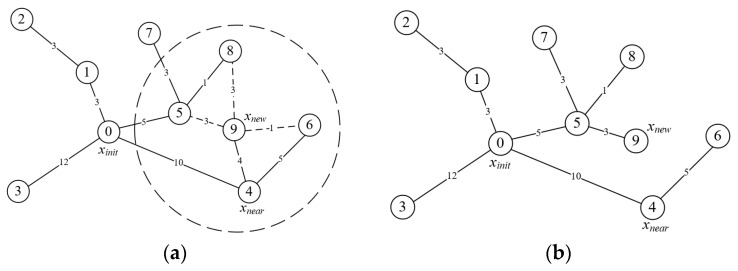
The process of reselecting the parent node: (**a**) the constrained search range when reselecting parent nodes; (**b**) the node situation after reselecting the parent node.

The RRT* algorithm enhances the RRT algorithm by incorporating improved strategies for parent node selection and reconnecting when sampling new nodes [23], which reduces the generated redundant paths and makes the planned paths better. The procedure for reselecting parent nodes is depicted in Figure 2.

With node $x_{new}$ as the center of the circle, form a constrained search range within the defined radius, as shown in Figure 2a. The neighboring nodes in it are used as candidates for the parent node of $x_{new}$. When solving for taking each candidate as a parent node, the cost from the starting point to $x_{new}$ is calculated and compared with the cost of the original path; the candidate node with the lowest cost is chosen as the new parent node. This results in the least costly path within the constrained radius size at this point in time, and the candidate node for the path at this point in time is selected in place of the original node [24]. The path cost with the candidate point as the parent node is shown in Table 1, and the parent node should be updated to node 5, as shown in Figure 2b.

**Figure 3:**
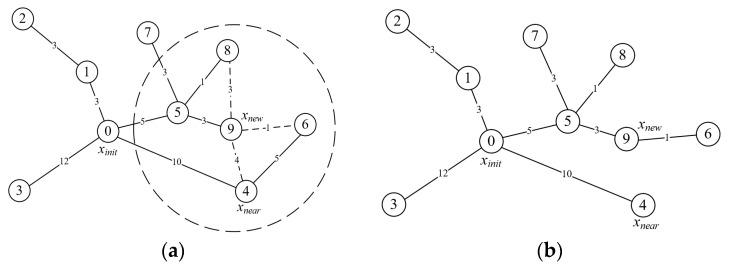
Reconnection process: (**a**) the constrained search range for reconnection; (**b**) the post-node situation after reconnection.

After completing the parent node reselection, a reconnection process is also required to prune the random tree [25], thereby further reducing the path length. Within the same constraints, as shown in Figure 3a, find all the neighboring nodes of node $x_{new}$ with $x_{new}$ as the parent of each neighboring node and find the cost of each path, i.e., if the cost is less than the cost of the path of the original parent of the neighboring node, update its parent to node $x_{new}$. The reconnection process is shown in Figure 3. From Table 2, the parent node of node 6 should be updated to node 9, as shown in Figure 3b.

Parent node reselection and reconnection strategies complement and interact with each other to significantly improve the path quality.

Although the parent node reselection and reconnection strategy in RRT* can improve the path quality, it increases the time cost and still has a slow convergence speed, which requires a large number of iterations to approach the better path. When dealing with a complex obstacle environment, especially in the case of a narrow or curved channel, there are some problems, such as unstable path quality, low algorithm efficiency, even planning failure, redundant nodes, and an unsmooth path, and there is still a lack of targeted sampling. Therefore, the improved strategy should be introduced into the RRT* algorithm according to Lemma 1.

**Lemma 1.**  (improving the efficiency of the RRT* algorithm): the improved RRT* algorithm reduces the number of invalid sampling points by introducing heuristic information or an optimization strategy, thus improving the efficiency of the algorithm.

### 2.2. Traditional Artificial Potential Field Method

The artificial potential field method views the vehicle motion space as a virtual force field space, with the target point on the vehicle producing gravitational force and the obstacle producing repulsive force. Therefore, the vehicle moves under the effect of these two forces.

Within the virtual realm of a force field, the attraction of a target point to a vehicle is directly proportional to the distance between them. The equation for the gravitational potential field function is provided below.

(1) $$U_{att}(d)=\frac{1}{2}k_{att}d^{2}(p,p_{g})$$

where $k_{att}$ is the gravitational potential field gain coefficient, p is the current location of the vehicle, $p_{g}$ denotes the position of the target point, and $d(p,p_{g})$ is a vector of magnitude representing the distance between the vehicle and the target point in the direction of the vehicle pointing to the target point. The gravitational force function is obtained from the above equation, as shown in the following equation.

(2) $$F_{att}(d)=k_{att}d(p,p_{g})$$

When the vehicle is out of its repulsive potential field’s range, the obstacle exerts no repulsive force. Only within the range of the repulsive potential field will the obstacle’s repulsive force affect the vehicle; the greater the distance between the two, the smaller the vehicle’s exposure to the repulsive potential energy value, and vice versa. The following equation illustrates the repulsive potential field function.

(3) $$U_{req}(d)=\left\{\begin{array}{c} \frac{1}{2}k_{rep}{\left(\frac{1}{d(p,p_{0})}-\frac{1}{d_{0}}\right)}^{2}\frac{1}{d^{2}(p,p_{0})},0\leq d(p,p_{0})\leq d_{0}\\ 0,d(p,p_{0})>d_{0} \end{array}\right.$$

where $k_{rep}$ is the repulsive potential field gain coefficient, p is the current position of the vehicle, $p_{g}$ denotes the position of the target point, and $d(p,p_{g})$ is a vector of magnitude representing the Euclidean distance between the vehicle and the obstacle in the direction of the vehicle pointing towards the obstacle. The repulsive force function on the vehicle can be calculated from the above equation as follows:

(4) $$F_{req}(d)=\left\{\begin{array}{c} k_{rep}(\frac{1}{d(p,p_{0})}-\frac{1}{d_{0}}),0\leq d(p,p_{0})\leq d_{0}\\ 0,d(p,p_{0})>d_{0} \end{array}\right.$$

In the process of driving, the vehicle will be affected by both the gravitational field and the repulsive field. The combined potential field to which it is subjected is as follows:

(5) $$U(d)=U_{att}(d)+\sum_{i=0}^{n}U_{rep}(d)$$

where n is the number of obstacles that have a repulsive effect on the vehicle.

From the combined potential field, we can obtain the combined force applied to the vehicle during its motion, and the combined force expression is given in the following equation:

(6) $$F(d)=F_{att}(d)+\sum_{i=0}^{n}F_{rep}(d)$$

However, the traditional artificial potential field method is prone to local minimum problems and unreachable targets. As a result, the unmanned car may fall into a “trap area” (where the resultant force on the vehicle is zero). For example, in the face of U-shaped obstacles, it is easy to fall into the local minimum area, so that the target point cannot be reached. It is also possible that the unmanned vehicle cannot reach the target position because the strong repulsive force will produce concussive behavior, which will eventually lead to the failure of path planning.

## 3. Improved RRT* Algorithm

### 3.1. Introducing the Concept of Artificial Potential Fields

#### 3.1.1. Introducing the Gravitational Component

**Figure 4:**
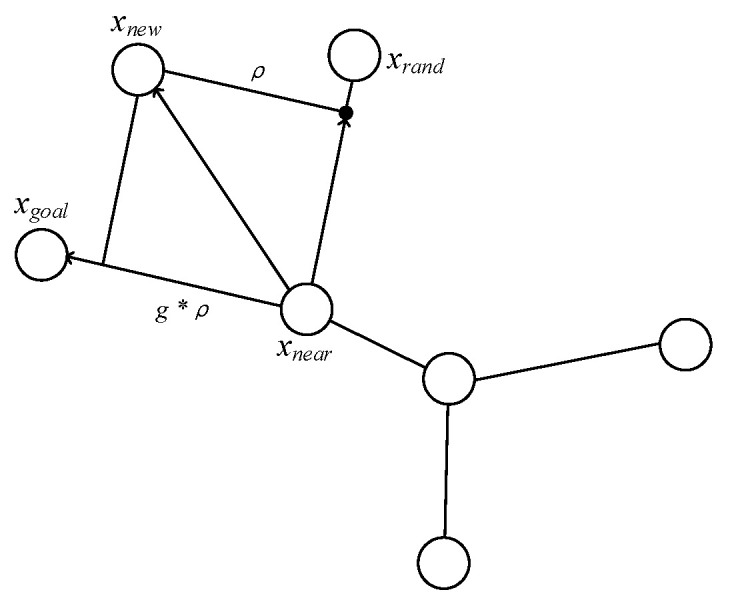
Random tree growth with increasing gravitational components.

In the RRT* algorithm, the gravitation between the random node and the target point is increased to guide the random tree to grow to the target point more rapidly and reduce the randomness. The schematic diagram is shown in Figure 4. In order to change the resultant force of the growth direction, the gravity function $G\left(n\right)$ is added to each new node n as follows:

(7) $$F_{1}(n)=Rd(n)+G(n)$$

where $F_{1}(n)$ is the function of the new node, Rd(n) is the random function of new nodes, and G(n) is the objective gravity function.

The gravity function G(n) is as follows:

(8) $$G(n)=\rho \times g\times \frac{x_{goal}-x_{near}}{\left|\left.x_{goal}-x_{near}\right|\right.}$$

where ρ is the step size, g is the gravity gain factor, $x_{goal}$ is the target position vector, $\left|x_{goal}-x_{near}\right|$ is the magnitude of the geometric distance between node $x_{goal}$ and node $x_{near}$.

Random function $Rd\left(n\right)$:

(9) $$Rd(n)=\rho \times \frac{x_{goal}-x_{near}}{\left|\left.x_{goal}-x_{near}\right|\right.}$$

#### 3.1.2. Introduction of Repulsive Force Component

**Figure 5:**
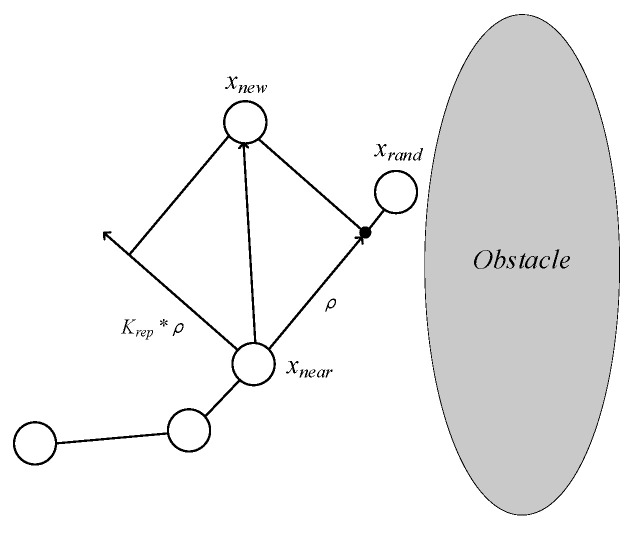
Random tree growth with increasing repulsion component.

When the RRT* algorithm expands the tree, it makes the random tree have a certain distance from the obstacle by increasing the repulsive component, as shown in Figure 5. The repulsive force component is added to the new nodes generated in the process of random tree expansion. Add the repulsion function T(n) at node n, as follows:

(10) $$F_{2}(n)=Rd(n)+T(n)$$

where $F_{2}(n)$ is the new node in the search process, Rd(n) is the random growth function, and T(n) is the repulsion function.

The obstacle repulsion function T(n) is as follows:

(11) $$T(n)=\left\{\begin{array}{c} 0,p(x)>p_{0}\\ \rho k_{rep}(\frac{1}{p(x)}-\frac{1}{p_{0}})\frac{1}{p{\left(x\right)}^{2}}\frac{\partial (x_{near}-x_{obstacle})}{\partial x_{near}},p(x)\leq p_{0} \end{array}\right.$$

where $k_{rep}$ is the repulsive force gain coefficient, p(x) is the shortest distance from $x_{rand}$ to the obstacle, $p_{0}$ is the distance affected by the obstacle on the node, and $x_{obstacle}$ is the position vector of the obstacle.

The new node generating function F(n) is as follows:

(12) $$F(n)=F_{1}(n)+F_{2}(n)$$

where $F_{1}(n)$ represents the new node function of the random tree in the gravitational field, and $F_{2}(n)$ represents the new node function of the random tree in the repulsion field. It is known that F(n) is not only determined by $x_{rand}$, which avoids random sampling of the RRT*.

### 3.2. Probabilistic Sampling Optimization

Because of the strong randomness in sampling, the RRT* algorithm often causes random trees to deviate from growth, which seriously affects the efficiency of the algorithm. It can be seen from Theorem 2 that in order to find a feasible path faster, the random tree of the algorithm can grow more purposefully, according to Lemma 1. Therefore, probabilistic sampling optimization is designed to improve target orientation and reduce redundant searches [26].

**Figure 6:**
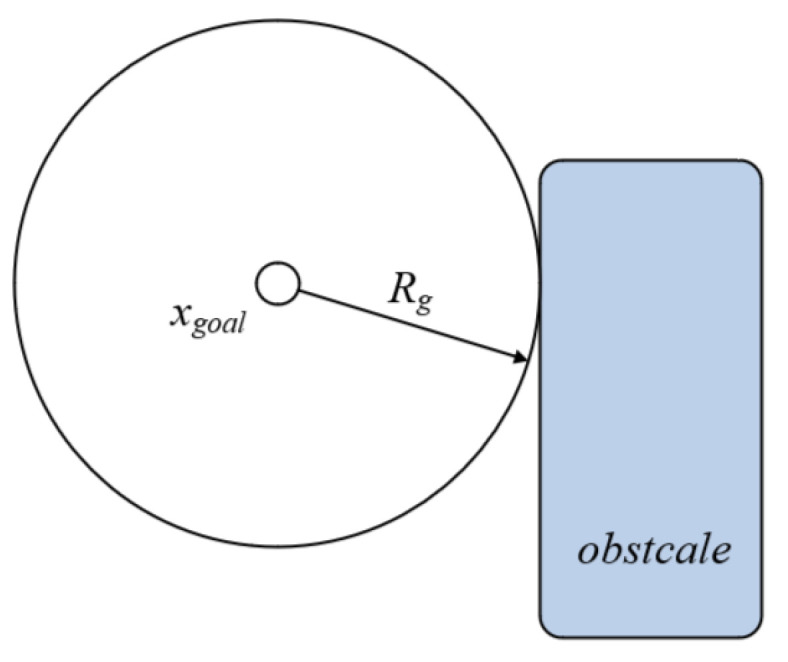
Schematic diagram of convergence region of target point.

Firstly, the obstacle-free area of the target point is determined, that is, the minimum distance from target $x_{goal}$ to all obstacles, which is called the convergence radius of target point $R_{g}$. As depicted in Figure 6, a region circle is generated with $R_{g}$ as the radius and $x_{goal}$ as the center, and the inner circle is the convergence region of the target point. Its characteristic is that when the random tree grows within this range, it can direct itself to the end point.

In the strategy of target bias, a target bias probability value $p_{target}$ is randomly generated by using the random number generating function, and then a random probability value p is obtained, which is greater than 0 and less than 1. When $p\geq p_{target}$ is obtained, then the sampling point is randomly generated. If $p<p_{target}$ is in the convergence region of the target point, the random extension tree grows near the target point with a certain probability by randomly generating a point as a sampling point. This method can accelerate the convergence rate to the target node. The sampling point-generating function is as follows:

(13) $$X_{rand}=\left\{\begin{array}{c} GoalArea(),p<p_{target}\\ Sample(),p\geq p_{target} \end{array}\right.$$

where the *GoalArea* function represents the random generation of a sampling point in the convergence region of the target point, the *sample* function represents the random generation of a sampling point in the global range, and $x_{rand}$ represents the randomly generated sampling point.

The probabilistic sampling optimization of the global path search is carried out [27]. When $p\leq p_{target}$, the target offset sampling is adopted, that is, a point is randomly generated in the target convergence domain as the sampling point. When $p_{target}<p\leq p_{goal}$, there is uniform random sampling in the search space.

**Figure 7:**
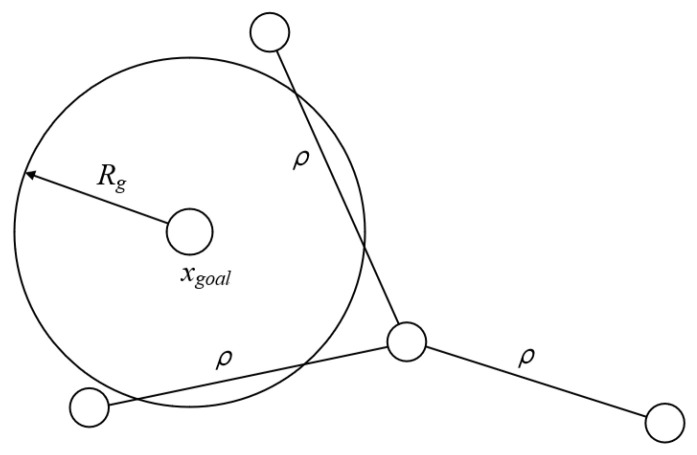
The limitation of the random tree near the target point.

When a new node coordinate $x_{new}$ is generated, it is determined whether the $x_{new}$ is in the target point convergence region. If so, the $x_{new}$ is directly connected to the terminal $x_{goal}$, but the step size ρ may be set to be larger than the target convergence radius $R_{g}$, causing the random tree to oscillate when it expands near the $x_{goal}$. This will cause the random tree to have limitations at the target point, as depicted in Figure 7.

In order to avoid this situation and make it get the optimal path faster, when $p\geq p_{goal}$ occurs, all of the random tree nodes are traversed to find the node $x_{k}$ with the lowest cost of $c_{min}$, as shown in the following formula:

(14) $$c_{min}=C^{k}+d\left(x_{k},x_{goal}\right)$$

where $C^{k}$ is the actual path cost from $x_{init}$ to $x_{k}$, and $d\left(x_{k},x_{goal}\right)$ is the Euclidean distance from node $x_{k}$ to target point $x_{goal}$. Connect $x_{k}$ and $x_{goal}$, then perform collision detection on the path from $x_{k}$ to $x_{goal}$. If no collision occurs, $x_{k}$ is directly used as the parent node of $x_{goal}$, and then the path is generated. If a collision occurs, it is changed to uniform random sampling, where $p_{target}$ and $p_{goal}$ are between 0 and 1.

In the initial target offset strategy, the target bias probability of the RRT* algorithm is fixed, and its adaptability to the scene is poor. In order to improve adaptability, a probability adaptive target bias strategy is designed to expand the tree growth combined with the gravity component constraints mentioned above to shorten the planning time [28].

**Figure 8:**
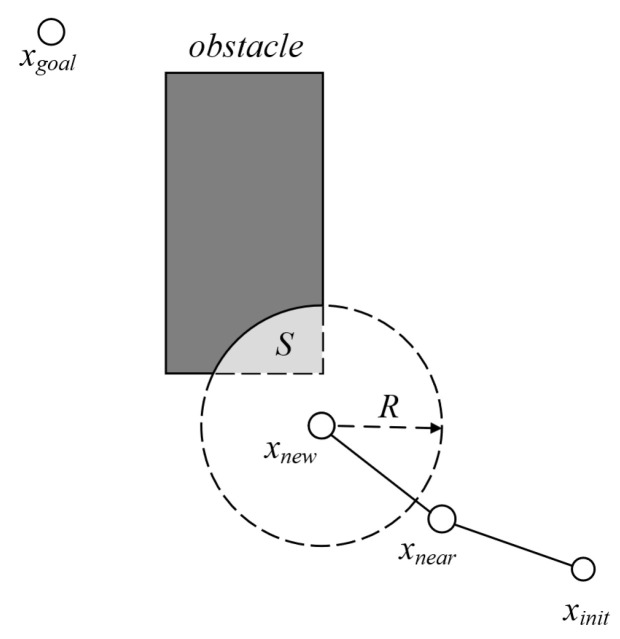
Construct the decision area.

Before generating a new sampling point, a decision circle is generated with the center of $x_{new}$ as the center and the connection between $x_{near}$ and $x_{new}$ as the radius R, as shown in Figure 8.

In Figure 8, $x_{new}$ is the center of the circle, R is the radius, and S is the area of the obstacle in the circle. The proportional value P can be obtained from the area value S, as shown in the following formula:

(15) $$P=\frac{S}{{\left(\pi *R\right)}^{2}}$$

Then change the scale value P to the target offset probability value $p_{target}$; the formula is as follows:

(16) $$p_{target}=1-e^{-\left(P*4.5\right)}$$

In the process of determining circle generation, the proportional value P will change with the change of radius R and occupied area value S, while the probability value $p_{target}$ will change with the change of proportional value P, so as to realize the self-adaptation of target bias probability. After the target bias probability value $p_{target}$ is obtained and compared with the random probability value p, sample according to the probability sampling optimization method mentioned above.

When the target offset sampling is adopted, the expansion tree grows to the target point, and the random node $x_{rand}$ will appear in the direction of the $x_{goal}$ and $x_{near}$ connections. Set the step size to ρ, and generate the formula for the new node $x_{new}$ as follows:

(17) $$x_{new}=x_{near}+\frac{x_{rand}-x_{near}}{\left\|x_{rand}-x_{near}\right\|}*\rho$$

When uniformly randomly sampled in the search space, the extension tree grows in the direction of the resultant force of random and gravitational components, so that the expansion of the new node will not deviate from the shortest path and enhance the goal bias of path sampling. The diagram of the new node is shown in Figure 4 above. The following formula will generate the new node $x_{new}$:

(18) $$x_{new}=x_{near}+\left(\frac{x_{rand}-x_{near}}{\left\|x_{rand}-x_{near}\right\|}*\rho +\frac{x_{goal}-x_{near}}{\left\|x_{goal}-x_{near}\right\|}*g*\rho \right)$$

### 3.3. Adaptive Step Size Strategy

In the process of global planning sampling, it can be seen from Theorem 1 that the algorithm should speed up the search speed, make its iterative progress faster, and improve the path quality. Therefore, in this improvement, the adaptive step size should be designed according to Lemma 1, so as to accelerate the sampling efficiency and improve the path quality. Different road environments need different steps. If the step size is too short, when there are few obstacles near the vehicle, or if the curvature of the road is small, the search takes a long time and reduces the search efficiency. If the step size is too large, there are many obstacles in the environment, or if the road has a large curvature, it may fall into a stagnant state and be unable to generate a feasible path. In order to further optimize the step size selection, an adaptive step size strategy is proposed. The step size ρ uses the number of obstacles near the vehicle and the curvature of the road so as to improve the adaptability of the vehicle to the environment. The formula for calculating curvature k is as follows:

(19) $$k=\frac{\ddot{y}}{{\left(1+{\left(\dot{y}\right)}^{2}\right)}^{\frac{3}{2}}}$$

where y is the road function. As the vehicle runs, there is continuous acquisition of vehicle location information and lane conditions, and the curvature of the lane is calculated.

The adaptive step size policy formula is as follows:

(20) $$\rho =\rho_{0}e^{\lambda \left(p-k-q\right)}$$

where $\rho_{0}$ is the initial step, p is the threshold of the number of obstacles, k is the curvature of the road, q is the number of obstacles, and λ is a value between 0 and 1.

When the number of obstacles around vehicle q and the road curvature k are zero, more than double the initial step size of $\rho_{0}$ is selected to generate new nodes. When the number of obstacles q is less than or equal to the threshold p and the curvature of the road is small, it means that the vehicle is driving on a road similar to a straight line and the number of obstacles around is small, and a new node is created by using a step close to the initial step size of $\rho_{0}$. When the number of obstacles q is greater than the threshold p, it shows that there are a large number of obstacles around the vehicle. When there is a large curvature on the road, it means that the road environment where the vehicle is located is a large curve. Consider that the vehicle cannot come into contact with obstacles; a new node is created by 0–1 times the initial step length of $\rho_{0}$. After the above process, the sampling step size can automatically change the applicable value in different road environments, improving the adaptability of the algorithm and enhancing the robustness of path planning.

When the target point is on the straight road and there are few obstacles nearby, a longer step size should be selected, but using a larger expansion step size will produce the oscillation phenomenon shown in Figure 7, resulting in an increase in path planning time and redundant nodes. In the optimization of probabilistic sampling, although it is possible to take $x_{goal}$ as the parent node near the target point, there is still probability. Therefore, it is necessary to further optimize the algorithm’s performance. The distance Distance between $x_{nearest}$ and $x_{goal}$ is calculated before each expansion. If Distance>ρ, the expansion step size remains the same; otherwise, the expansion step size is adjusted to the Distance more suitable for growing near $x_{goal}$, and the oscillation problem is solved.

### 3.4. Global Path Post-Processing

After the above-mentioned improvement strategies, the algorithm in this paper can quickly plan the initial path, but there may still be some tortuous paths and a large number of unnecessary nodes [29]. First of all, for the redundant paths, the triangle principle is used to design the optimization node strategy, and then for some of the tortuous paths, the constraint based on the minimum turning radius is established [30].

#### 3.4.1. Node Pruning Strategy

**Figure 9:**
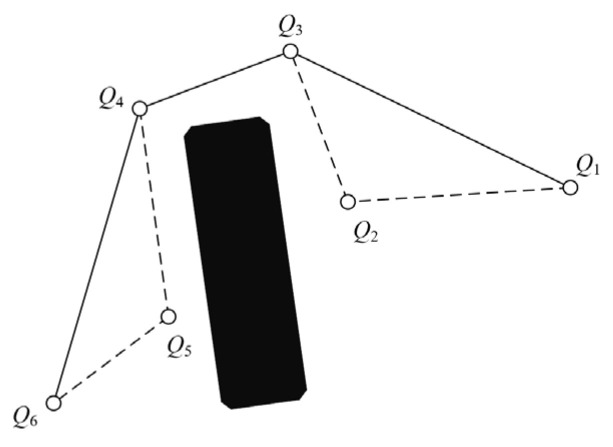
Schematic diagram of node branch pruning strategy.

The principle of the node pruning strategy is depicted in Figure 9. The dashed line represents the initial path planned by the improved RRT* algorithm without incorporating a node pruning strategy. The planning path is formed by the nodes in the initial node set $\left\{Q_{1},Q_{2},Q_{3},Q_{4},Q_{5},Q_{6}\right\}$, where $Q_{1}$ and $Q_{6}$ are the starting point and target point, respectively. After introducing the node pruning strategy, take the target point $Q_{6}$ as the root node, and each node in the initial node set is connected successively based on $Q_{6}$, and collision detection is carried out. It is found that line segment $Q_{6}Q_{1}$, line segment $Q_{6}Q_{2}$, and $Q_{6}Q_{3}$ will collide with obstacles, but there is no collision when $Q_{6}$ is directly connected to $Q_{4}$, so $Q_{4}$ is added to the set of optimized nodes. Then, taking $Q_{4}$ as the starting point of collision detection, $Q_{4}$ can only connect with $Q_{3}$ without collision, so $Q_{3}$ is added to the set of optimized nodes. Connect each node in the original path in turn and carry out a new round of collision detection. We can know that when $Q_{1}$ can be directly connected to $Q_{3}$, when the starting point is added to the optimized node set, it indicates that the node reconnection ends, that is, $Q_{1}$ is put into the optimized node set. According to the order of the nodes in the optimized node set, the optimization path is obtained. The path is depicted by the solid line in Figure 9. Compared with the original path, it is observed that the optimized path features a substantial reduction in node count and effectively decreases the path’s length cost.

#### 3.4.2. Node Optimization

**Figure 10:**
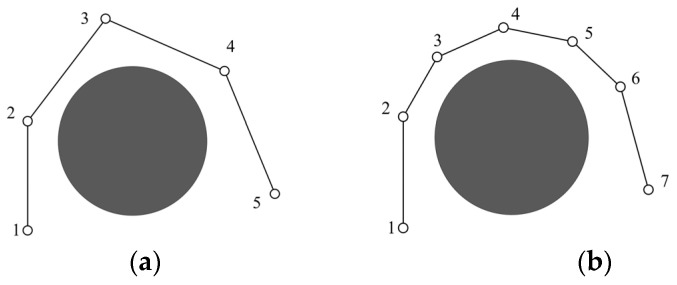
Schematic diagram of node optimization strategy: (**a**) before node optimization; (**b**) after node optimization.

After the path is treated by the node branch and shear, the joint angle of part of the path cannot meet the vehicle dynamics constraint, so it is necessary to optimize the route with too large a bend angle to ensure that the turning angle at the turning point of the path satisfies the vehicle kinematic constraints [31]. And the continuous folding angle is optimized to make the turning angle more uniform and stable. The process is illustrated in Figure 10.

As shown in Figure 10a, in the planned path, the angle between node 2 and node 3 and node 4 and node 5 does not conform to the vehicle kinematic constraint, so it needs to be optimized. This problem is solved by adding auxiliary nodes, such as nodes 3, 4, and 5 in Figure 10b, which flatten the initial angle so as to meet the minimum turning radius constraint of the vehicle.

## 4. Improved Artificial Potential Field Method

### 4.1. Obstacle Avoidance Constraint

**Figure 11:**
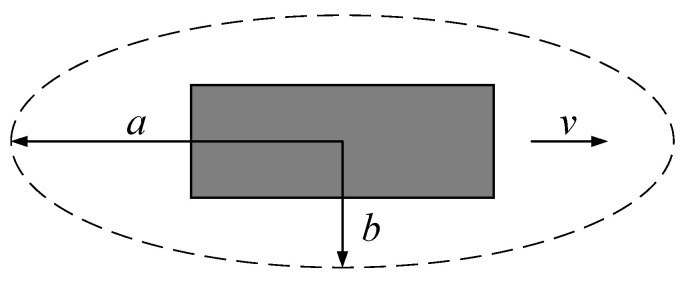
Schematic diagram of environmental obstacles.

When modelling obstacles in the road environment, the threat in the longitudinal direction is generally greater than that in the horizontal direction, so the longitudinal distance between the main vehicle and the environmental obstacles should be fully considered when establishing obstacle avoidance constraints. Therefore, the surrounding obstacles are modelled as ovals. The major axis of the ellipse corresponds to the longitudinal axis of the obstacle, and the short axis of the ellipse corresponds to the horizontal axis of the obstacle. Considering that the obstacle has speed, this will impact the main vehicle’s obstacle avoidance performance. Therefore, a dynamic ellipse model of environmental obstacles, which can change with speed, is designed. The long axis length of the ellipse is associated with the velocity parameter. Thus, velocity is directly related to the length of the dynamic ellipse’s major axis, indicating that a larger influence area corresponds to a lower collision probability [32]. Environmental obstacles are depicted, as illustrated in Figure 11.

The major and minor axes of the dynamic ellipse are denoted as follows:

(21) $$a=\frac{L}{2}+k_{a}+k_{r}v$$

(22) $$b=\frac{W}{2}+k_{b}$$

where L represents the length of the obstacle, W represents the width of the obstacle, v denotes the speed of the obstacle, $k_{a}$ and $k_{b}$ are horizontal and vertical security redundancy distances, and $k_{r}$ is the safety redundancy time.

**Figure 12:**
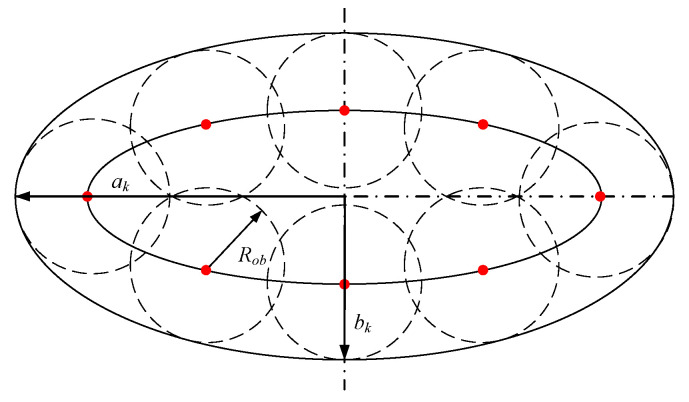
Extended ellipse.

In the above modelling, the collision can be judged by judging whether there is an overlap between the main vehicle and the ellipse boundary. The boundary of the ellipse is divided into many circles, and several circles are made on the boundary line of the ellipse with a width of 1/2 of the vehicle as the radius, thus forming a new ellipse, that is, the extended ellipse. It is equivalent to increasing the collision range by half a vehicle’s width on the basis of the original obstacle ellipse. The distance between the vehicle and the obstacle is increased, and the path safety is improved. The extended ellipse is shown in Figure 12.

The major and minor axes of an extended ellipse are expressed as follows:

(23) $$a_{k}=\frac{L}{2}+k_{a}+k_{r}v+R_{ob}$$

(24) $$b_{k}=\frac{W}{2}+k_{b}+R_{ob}$$

When the vehicle is driving on the planned path, the point mass model is used to simplify the vehicle, and collision detection is performed by assessing the spatial relationship between the point mass model and the extended ellipse. If the center of mass lies within or on the extended ellipse, a collision is deemed to have occurred, and the collision constraint is not met. Conversely, if the center of mass is located outside the extended ellipse, no collision is considered to have taken place, and the collision constraint is satisfied.

### 4.2. Road Repulsion Potential Field

During main car operation on a two-lane road, the lane line and lane boundary should cause repulsion to the moving vehicle, and when there are no obstacles in the road, the vehicle should not leave the current lane unless it is necessary to change lanes when obstacles are encountered. Vehicles are not allowed to press the line or cross the road boundary to avoid traffic accidents.

In the road repulsion potential field model design, ensuring that the repulsion potential field at the lane boundary is the highest serves to confine the vehicle within the lane boundary [33]. While preserving the boundary repulsion potential field, the lane repulsion potential field is incorporated. To ensure smooth lane changes for the main vehicle, the maximum value of the lane’s repulsive potential field must be lower than that of the obstacle. A novel road repulsion potential field is formulated to prevent vehicle lane changes in the absence of obstacles. The repulsion potential field for a vehicle travelling along the centerline of the current lane is minimized to constrain the vehicle’s ability to remain in the lane’s centerline. The road repulsion potential field $U_{road}$ is obtained by superposing the lane line potential field $U_{l}$ and the road boundary potential field $U_{r}$. The formula for the road repulsion potential field $U_{road}$ is expressed as follows:

(25) $$U_{road}=U_{l}+U_{r}$$

The lane line potential field $U_{l}$ can make the vehicle keep a certain distance from the lane and make it move towards the center of the lane. It should be guaranteed to overcome the potential field when encountering obstacles, which requires that the lane potential field be small enough to ensure driving safety. The lane line potential field established in this paper is expressed by the following formula:

(26) $$U_{l}=\sum_{i}^{N_{ls}-1}U_{l,i}$$

(27) $$U_{l,i}=A_{l}\exp\left(-\frac{{\left(y-y_{c,i}\right)}^{2}}{2\sigma^{2}}\right)$$

where $A_{l}$ represents the intensity coefficient of the lane potential field, $y_{c,i}$ is the central position of the Gaussian function, the i lane coordinate is generally set on the centerline of the lane, and σ determines the range of the potential field, that is, the sensitivity of the vehicle to the lane centerline.

**Figure 13:**
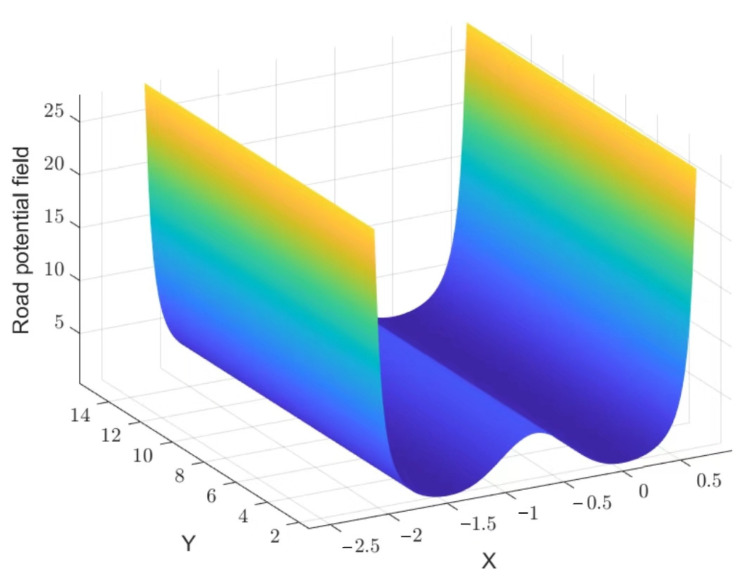
Road repulsion potential field diagram.

Set the road boundary potential field $U_{r}$ to infinity to limit vehicle crossing, as shown in the $U_{r}$ expression:

(28) $$U_{r}=\sum_{j}^{N}U_{r,j}$$

(29) $$U_{r,j}=\frac{1}{2}\eta {\left(\frac{1}{y-y_{0,j}}\right)}^{2}$$

where η is the potential field factor of the boundary road, $y_{0,j}$ is the y coordinate of the j road boundary, and N represents the number of road boundaries. Figure 13 shows the road potential field diagram of the Dow repulsion.

### 4.3. Increase Distance Factor

To address the issue that the target point is unreachable, it is necessary to optimize the repulsion function and add the distance factor $d_{g}^{2}$ [34]. The improved repulsive potential field function is as follows:

(30) $$U_{rep}\left(d,\alpha \right)=\left\{\begin{array}{l} \frac{1}{2}k_{\alpha }k_{rep}{\left(\frac{1}{d\left(p,p_{0}\right)}-\frac{1}{d_{0}}\right)}^{2}d_{g}^{2},0\leq d\left(p,p_{0}\right)\leq d_{0}\\ \;\;\;\;\;\;\;\;\;\;\;\;\alpha \in \left(0,\pi \right)\\ \;\;\;\;\;0\;\;\;\;\;,d\left(p,p_{0}\right)>d_{0} \end{array}\right.$$

After introducing the distance factor $d_{g}^{2}$, the repulsion from the obstacle and the attraction from the target point will both diminish to zero only when the vehicle reaches the target point. This resolves the issue of the target being unreachable when the obstacle is in close proximity to the target point. However, when there are multiple obstacles, they may lead to local minima because their repulsion and gravity may be in the opposite direction. Therefore, the repulsion function of the obstacle is improved, and a repulsion term pointing to the target point is added. The enhanced repulsion function is defined as follows:

(31) $$F_{rep}\left(d,\alpha \right)=\left\{\begin{array}{c} F_{rep1}+F_{rep2},0\leq d\left(p,p_{0}\right)\leq d_{0}\cap \alpha \in \left(0,\pi \right)\\ 0,d\left(p,p_{0}\right)>d_{0} \end{array}\right.$$

**Figure 14:**
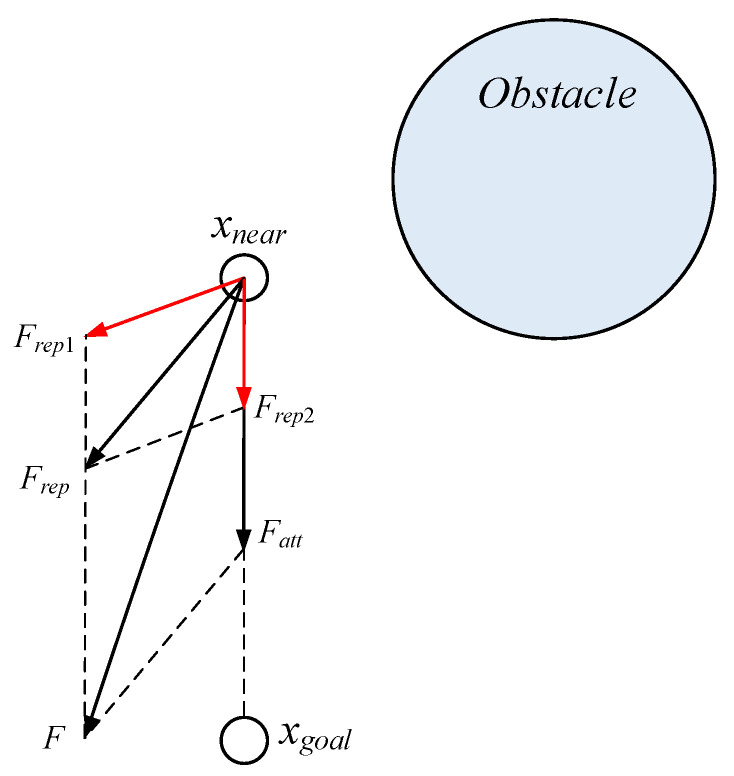
The force of the vehicle after optimizing the repulsion function.

The $F_{rep1}$ and $F_{rep2}$ expressions are as follows:

(32) $$F_{rep1}=k_{\alpha }k_{rep}\left(\frac{1}{d\left(p,p_{0}\right)}-\frac{1}{d_{0}}\right)d_{g}^{2}$$

(33) $$F_{rep2}=\frac{k_{\alpha }}{2}k_{rep}\left(\frac{1}{d\left(p,p_{0}\right)}-\frac{1}{d_{0}}\right)d_{g}$$

where $d_{0}$ represents the range of the obstacle’s repulsive potential field, d denotes the distance between the obstacle and the vehicle, $d_{g}$ represents the distance from the vehicle to the target point, $k_{rep}$ is the repulsive gain coefficient, $k_{\alpha }$ is the repulsive potential field constraint factor, $F_{rep1}$ is the repulsive force of the obstacle pointing to the vehicle, and $F_{rep2}$ is directed from the vehicle to the target point. Figure 14 shows the force analysis of the improved vehicle.

### 4.4. Virtual Target Point

**Figure 15:**
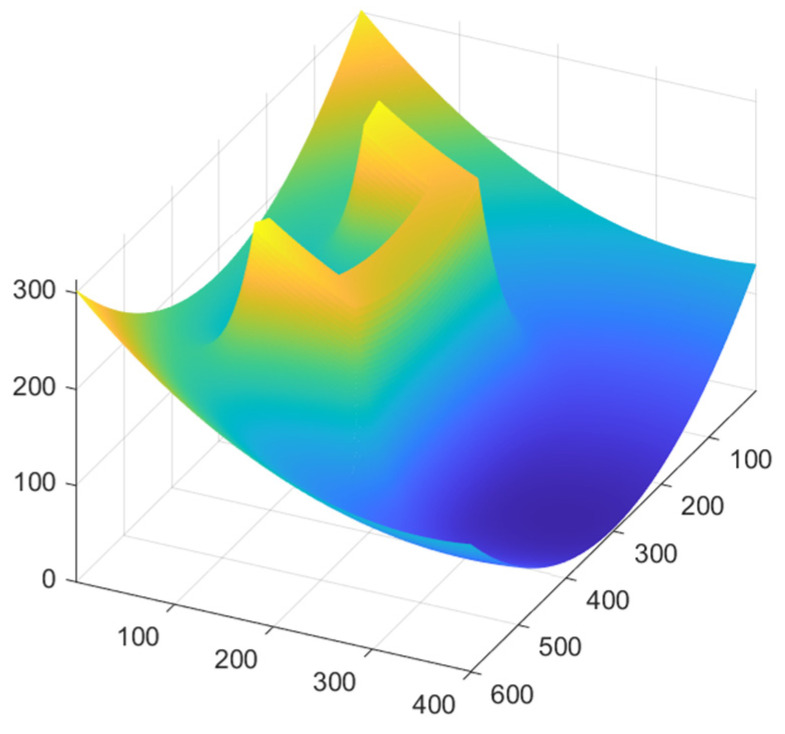
The general potential field near a U-shaped obstacle.

In the artificial potential field method, the vehicle moves from the high potential field to the low potential field, and the target point is the global minimum point of the potential field, so the vehicle should stop moving at the target point. When there are U-shaped obstacles in the road, the interior is the local minimum region, and the global minimum is the target point. Therefore, the minimum point will not only be at the target point; in this environment, the vehicle driving in the direction of low potential energy will enter the local minimum area of the U-shaped obstacle, which will cause the vehicle to stop or oscillate around this area. The overall potential field featuring a U-shaped obstacle is depicted in Figure 15.

**Figure 16:**
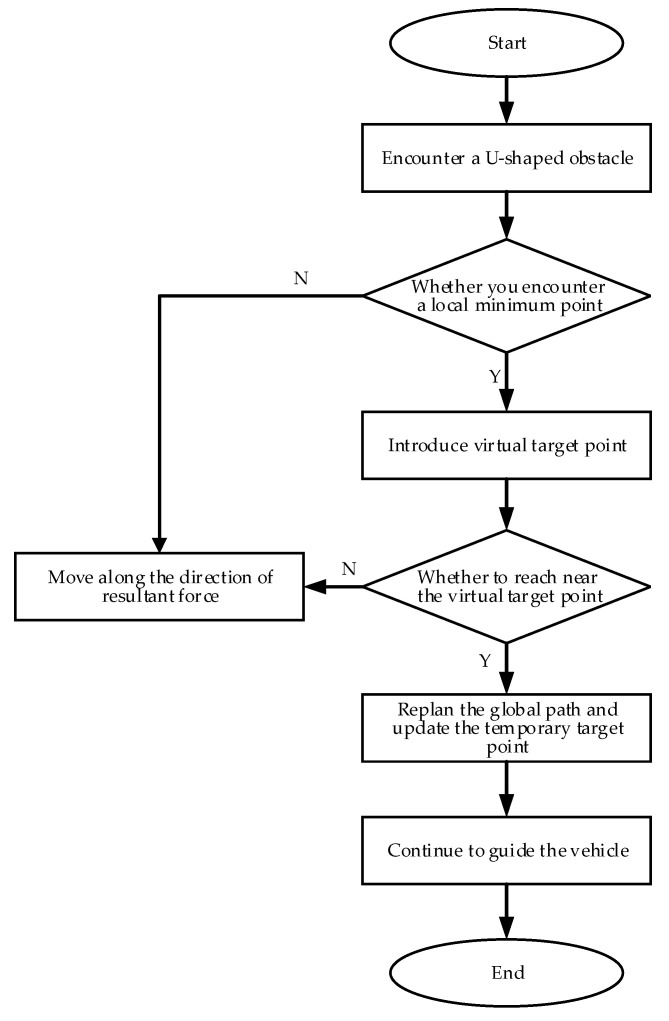
The processing flow of U-shaped obstacles.

To address this issue, a virtual target point is introduced to steer the vehicle away from obstacles. The potential field is adjusted to improve the passing capacity of the vehicle near the local minimum so as to plan a more reasonable path. When setting the virtual target point, the point is too far from the obstacle, which will lead to the planned path being too long, resulting in an increase in the amount of calculation. The point too close to the obstacle may reduce the safety of the path or even collide with the obstacle. The processing flow for encountering a U-shaped obstacle is shown in Figure 16.

Before introducing the virtual target point near the obstacle, first of all, judge whether the vehicle meets the local minimum point or not, and the discrimination condition is as follows:

(34) $$\left|F_{att}+\sum_{j=1}^{n}F_{rep,j}\right|<\varepsilon$$

(35) $$\left|x-x_{a}\right|<\alpha s_{a}$$

where j is the number of obstacles, ε is a small positive number, α is a positive number between 0 to 1, $x_{a}$ is a certain state of the vehicle, and $s_{a}$ represents the total distance of the vehicle from $x_{a}$ to the current position x in the process of vehicle movement.

When the determination condition is established, it means that the virtual resultant force on the vehicle is close to 0, and the vehicle has moved for a long distance but its displacement is very small, so it can be considered that the vehicle stops or oscillates around the local minimum [35]. When introducing the virtual target point, the role of the target gravitational potential field is ignored until the vehicle successfully reaches the virtual target point, so as to get rid of the predicament of falling into local optimization in the U-shaped obstacle. The virtual target point is selected where the distance from the obstacle is $L_{2}$ longitudinally and $L_{1}$ horizontally. The calculation method is as follows:

(36) $$L_{1}=\lambda a$$

(37) $$L_{2}=\mu b$$

(38) $$L=\sqrt{L_{1}^{2}+L_{2}^{2}}$$

where λ and μ are the distance expansion coefficients. According to the danger degree of the obstacle, the safe distance can be adjusted dynamically by changing the value of λ.

Through the above formula, the potential field function can be obtained:

(39) $$U_{vir}=k_{vir}\sqrt{{\left|x-x_{0}-L\right|}^{2}+{\left|y-y_{0}\right|}^{2}}$$

where $k_{vir}$ represents the coefficient representing the potential energy increase of the virtual target point.

**Figure 17:**
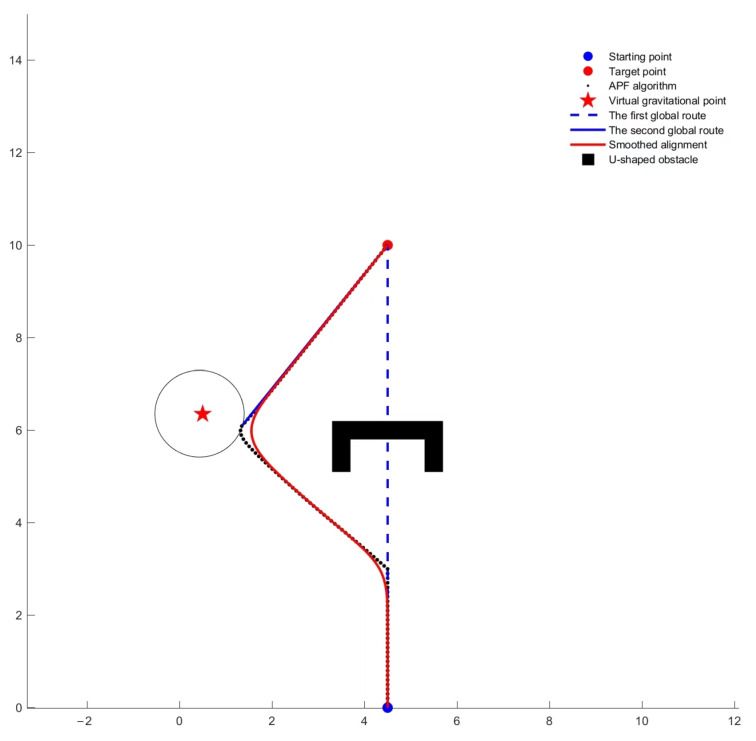
Virtual target point selection method.

Following the identification of the local minimum point, the vehicle becomes influenced by the potential field of the virtual target point. Considering that the target may be unreachable, a circle with a radius of $R_{v}$ is established as the virtual target area, with the virtual target point as the center. Guided by the global path, the vehicle encounters a U-shaped obstacle and introduces the virtual target point. When the vehicle runs into the area of the virtual target point, it will deactivate the virtual target point and proceed to utilize either the original target point or the updated target point until reaching the global target point. The virtual target point selection method is shown in Figure 17.

On the road, the vehicle is mainly affected by the gravitational potential field, the repulsive potential field, and the road potential field. If the virtual target point is introduced, all the potential fields in the road environment are shown as follows:

(40) $$U_{all}=U_{att}+U_{rep}+U_{road}+U_{vir}$$

### 4.5. Elliptical Groove Treatment

**Figure 18:**
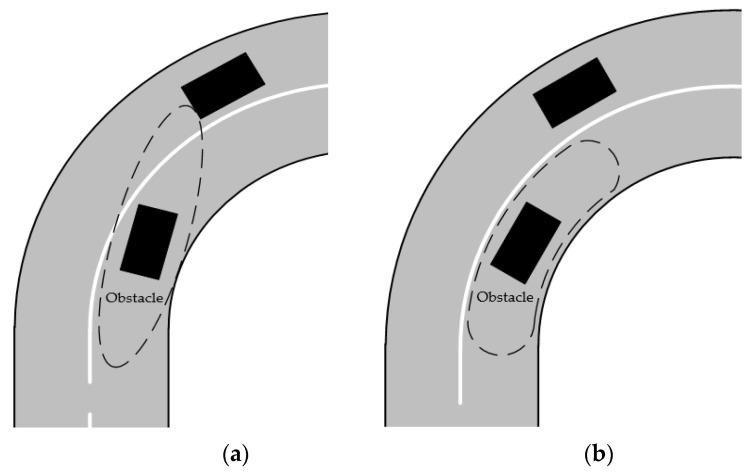
A comparison diagram of elliptical groove treatment: (**a**) a schematic diagram of the limitations of the repulsive domain of elliptical obstacles; (**b**) a schematic diagram of an elliptical groove.

On structured roads, obstacles typically pose a greater threat in the longitudinal direction than in the lateral direction [36], so this paper uses the obstacle avoidance constraint mentioned in Section 3.1 for the obstacle model. However, when driving on a curved road, the extended elliptical boundary of the obstacle cannot be deflected by the curvature of the road, so it will affect the trajectory of the main vehicle in the lane, resulting in unreasonable planning and even local minimums, as depicted in Figure 18a.

To address this issue, the extended ellipse boundary is processed by an elliptical groove by considering the curvature of the road. The center of curvature of the road is taken as the center of the two long boundaries of the elliptical groove, and the bending degree of the two boundaries of the elliptical groove is consistent with the bending degree of the lane line, and then the two long boundaries are closed and connected with two small arcs. Finally, an elliptical groove is formed, as depicted in Figure 18b.

## 5. Fusion Algorithm

### 5.1. Analysis of the Limitation of the Algorithm

In addition to the local minimum situation encountered by the artificial potential field method described above, the target is unattainable and there is the problem of facing U-shaped obstacles. There are also algorithmic limitations caused by road conditions.

**Figure 19:**
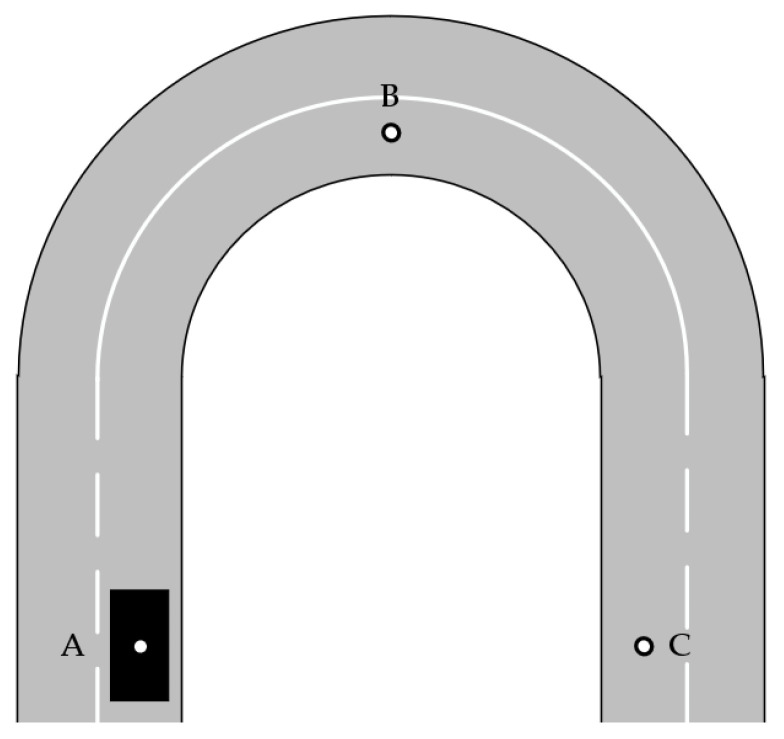
A schematic diagram of the limitations of the turning road.

As the distance between the vehicle and the target point increases, so does the gravitation in the potential field. Affected by the road conditions, when driving on the turning road, A is the starting point and C is the target point. The distance AC between the main car A and the target point C is less than the distance BC between the point B on the way and the target point C. Therefore, the potential field of point B will be larger than that of point A, and it is impossible to travel from the low potential field to the high potential field in the APF potential field, so there will be a local minimum, as illustrated in Figure 19.

In the actual driving scene, the environment is usually partially known, but there are often unknown obstacles. Therefore, path planning needs to consider global and local factors. Global path planning algorithms can usually find feasible paths in the whole environment but may not be able to deal with environmental changes and unknown obstacles. The local path planning algorithm can usually quickly respond to environmental changes and obstacle movement, but it only considers local factors, is likely to encounter local optimization, and so on. Therefore, as a local path planning algorithm, the artificial potential field method often falls into the local minimum point in a complex environment and cannot reach the target point smoothly due to insufficient global information. As a global algorithm, the RRT* algorithm is effective in static path planning when the road environment information is known. However, it lacks real-time obstacle avoidance capabilities when confronted with unknown obstacles.

### 5.2. Algorithm Fusion Strategy

To simultaneously accomplish global path optimization and local avoidance of unknown obstacles, this paper proposes a hybrid path planning approach integrating enhancements to the RRT* algorithm and the artificial potential field method. The RRT* algorithm is improved by using the strategies of probability sampling optimization, adaptive step size, and path post-processing, and the artificial potential field concept is introduced to expedite the discovery of the global path within the road environment. The artificial potential field method is a local obstacle avoidance algorithm for the real-time generation of obstacle avoidance paths that realizes path planning according to the motion state and environment information of the controlled object. Therefore, combining the improved RRT* algorithm and the enhanced artificial potential field approach suggests a fusion algorithm, which can combine the benefits of both global and local path planning algorithms. Before applying the artificial potential field method to dynamic path planning, the improved RRT* algorithm is used for the global path planning of static known environment roads, and the global path node sequence is extracted.

**Figure 20:**
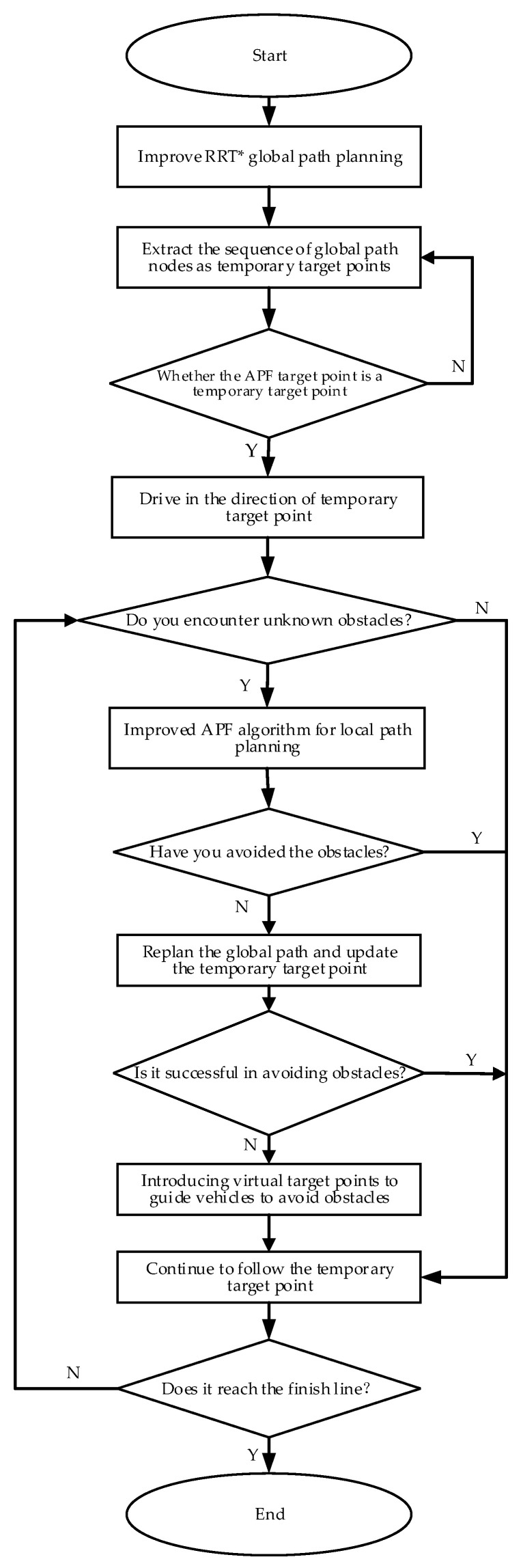
Fusion algorithm flow.

The initial and final points in the node sequence also serve as the starting and ending points for vehicle path planning. Make each node of the node sequence a temporary target point, and when the vehicle is driving along the path, the enhanced artificial potential field method is used to guide the vehicle by taking each node in the node sequence as the target point in turn. Once the vehicle reaches a node, the subsequent node is designated as a temporary target point until the vehicle reaches the global target point. When unknown obstacles are encountered in the driving process, the enhanced artificial potential field method is employed to locally plan the path, thereby accomplishing obstacle avoidance. Upon encountering a local minimum value, the improved RRT* global path is automatically re-planned, or the virtual target point is introduced to update the temporary target point position and direct the vehicle away from the local minimum region, thus solving the local minimum problem of the artificial potential field method and enhancing the vehicle’s obstacle avoidance performance. The final fusion algorithm’s workflow is depicted in Figure 20.

### 5.3. Path Smoothing Strategy

After the above path optimization, the path produced by the RRT* algorithm exhibits a turning angle, and the turning angle is too large, which is not suitable for vehicles. The Bezier curve only needs a few control points to generate a more complex, smooth curve [37]. The smooth processing of the path is realized by fitting the points on the path using the n-order Bezier curve. The vehicle can run smoothly.

Given points *P*_0_, *P*_1_, … *P_n_*, the nth-order Bezier curve is as follows:

(41) $$P(t)=\sum_{i=1}^{n}P_{i}B_{iN}(t),t\in [0,1]$$

where $P_{i}$ is the vertex coordinate, $B_{iN}(t)$ represents the *n*-degree Bernstein polynomial, and its basis function is as follows:

(42) $$B_{iN}(t)=\sum_{i=0}^{n}\left(\begin{array}{c} n\\ i \end{array}\right){\left(1-t\right)}^{n-i}t^{i}$$

where $\left(\begin{array}{c} n\\ i \end{array}\right)=\frac{n!}{i!(n-i)!}$; Formula (42) represents the n-th order formula of the Bezier curve determined by point $P_{0}、P_{1}\ldots P_{n}$; $P_{0}$, and $P_{n}$ are the starting point and ending point, respectively; and $P_{1}\ldots P_{n-1}$ is called the intermediate control point. Each Bezier curve is defined by a set of intermediate control points. In order to make the connection of a multi-segment curve smooth, the end control point and the first end control point of the adjacent segment should be selected so that the tangent direction and curvature of the connection point are continuous and a smooth path can be generated [38].

## 6. Simulation Analysis

In order to verify whether the improved RRT* algorithm and the fusion algorithm can search the target point and generate the path more effectively at the starting point of the space, the simulation experiments of the improved RRT* algorithm and the fusion algorithm are carried out on the turning road. In order to further verify the performance of the fusion algorithm, the simulation experiments are carried out in a more complex S-bend. The computer is configured with a Windows 11 operating system, an Intel (R) Core (TM) i5-8300H CPU in Dell, Texas, USA, a main frequency of 2.30 GHz, and a running memory of 16.0 GB. The simulation environment is built in the MATLABR2022b environment, and the simulation experiments of the improved RRT* algorithm and fusion algorithm are realized. In order to compare the experimental results, the simulation parameters of each algorithm are unified. The following are the simulation experiment condition settings and parameter selections:

The size of the simulation map in turn is 15 × 16. The starting node is (0, 0), and the target node is (9, 0). The size of the simulation map in the S-bend is 26 × 32. The starting node is (0, 0), and the target node is (21, 0). The white dotted line and the white solid line are the lanes that can be crossed by vehicles and the lanes that cannot be crossed by vehicles. The grey road area is represented as a safe area, and the black boundary on both sides means that vehicles are not allowed to touch the road boundary. The black rectangular object and the black U-shaped object are unknown obstacles and U-shaped unknown obstacles, respectively, and the red dotted line around the rectangular obstacle is the influence area of the obstacle. The initial step size, the initial target bias probability, the gravitational gain coefficient, the repulsion gain coefficient, the repulsive force range, the maximum number of nodes, and the maximum number of iterations are 0.5, 1.05, 1.15, and 1.25. The maximum number of nodes is 3000, and the maximum number of iterations is 7000. The position of the starting point is represented by a blue solid dot, while the position of the target point is represented by a red solid dot. Considering the randomness of RRT series algorithms, 50 simulation experiments are carried out on each algorithm, and the effectiveness of the improved algorithm is verified by comparing and analyzing the four performance indexes, namely, the average path length, the average number of nodes, the average simulation time, and the average number of iterations.

### 6.1. Simulation Analysis of Improved RRT* Algorithm

This simulation experiment is divided into Experiments 1 and 2. Experiment 1 is the simulation experiment of the improved RRT* algorithm before path post-processing, and Experiment 2 is the simulation experiment of path post-processing.

#### 6.1.1. Experiment 1

**Figure 21:**
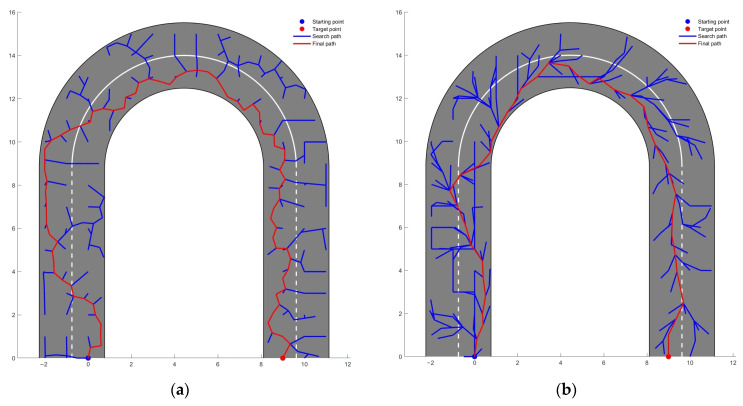

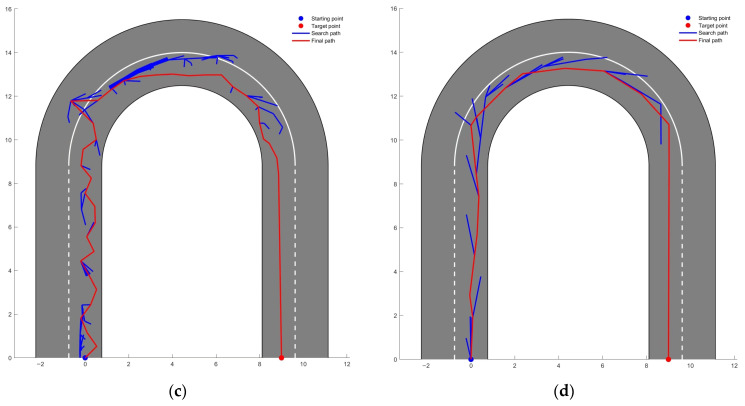
Comparison diagram of improved RRT* algorithm: (**a**) simulation path of traditional RRT algorithm; (**b**) RRT* algorithm simulation path; (**c**) simulation path of traditional improved RRT algorithm; (**d**) this paper improves RRT* algorithm simulation path.

To evaluate the effectiveness of the improved RRT* algorithm proposed in this paper, an analysis was conducted. Simulation experiments were conducted on the conventional RRT algorithm, the RRT* algorithm, and the proposed improved algorithm outlined in this paper. Among them, the traditional improved RRT algorithm, which introduces the concept of potential fields, is used to compare with the improved algorithm in this paper, which is commonly used in the improvement method of the RRT algorithm. The above four RRT algorithms are simulated, respectively, and the average path length, average number of nodes, average simulation time, and average number of iterations are compared and analyzed. To further confirm the efficacy of the enhanced algorithm, the improved algorithm is simulated and compared with the Dijkstra algorithm, the ant colony algorithm, the A* algorithm, and the improved A* algorithm, and the four performance indicators of each algorithm are analyzed. Figure 21 below displays the simulation outcomes of the aforementioned four algorithms. The solid blue line is the search path, and the red solid line is the final search path. Table 3 shows the comparison of the experimental data for the average path length, average number of nodes, average simulation time, and average number of iterations of the four algorithms.

Compared to the traditional RRT algorithm, the improved RRT* algorithm presented in this paper reduces the average path length by 14.37%, 4.64%, and 4.19% for the RRT* algorithm and the traditional improved RRT algorithm. The reduction in path length is not obvious. This occurs due to the excessive randomness inherent in the RRT algorithm, and there will still be redundant nodes. Moreover, the algorithm enhanced in this paper has not yet undergone the post-processing stage so far in the simulation experiment. However, in terms of the average number of nodes, compared with the RRT algorithm, the RRT* algorithm, and the traditional enhanced RRT algorithm, they were reduced by 85.19%, 71.43%, and 77.47%, respectively. This suggests that the enhanced RRT* algorithm significantly impacts the optimization strategy for redundant path branches, greatly reducing the number of redundant nodes. In terms of average simulation time, the average simulation time is reduced by 95.91%, 93.98%, and 45.98%, respectively, with the RRT algorithm, the RRT* algorithm, and the traditional improved RRT algorithm. At the same time, because the RRT algorithm is a random search algorithm, it also notably diminishes the path’s randomness attributed to time cost and can find the global search path more quickly. In terms of the average number of iterations, compared to the RRT algorithm, the RRT* algorithm, and the traditional improved RRT algorithm, the average number of iterations is reduced by 95.46%, 95%, and 51.48%, respectively, and the average number of iterations is significantly reduced, indicating that the path generation speed of the enhanced algorithm is faster, the time cost is lower, and the randomness of the RRT algorithm is reduced. Combined with Figure 21d, it can be seen that while ensuring a safe distance at the road boundary, the improved RRT* algorithm can generate the path more quickly and accurately, and the improved RRT* algorithm significantly reduces redundant nodes in the path, minimizes lateral fluctuations, and enhances path stability. Observation of Figure 21a–c shows that it is obvious that these algorithms have many redundant nodes, have a large invalid search range, and even extend to the opposite lane. The performance indicator comparison of the four algorithms is presented in Table 3.

**Figure 22:**
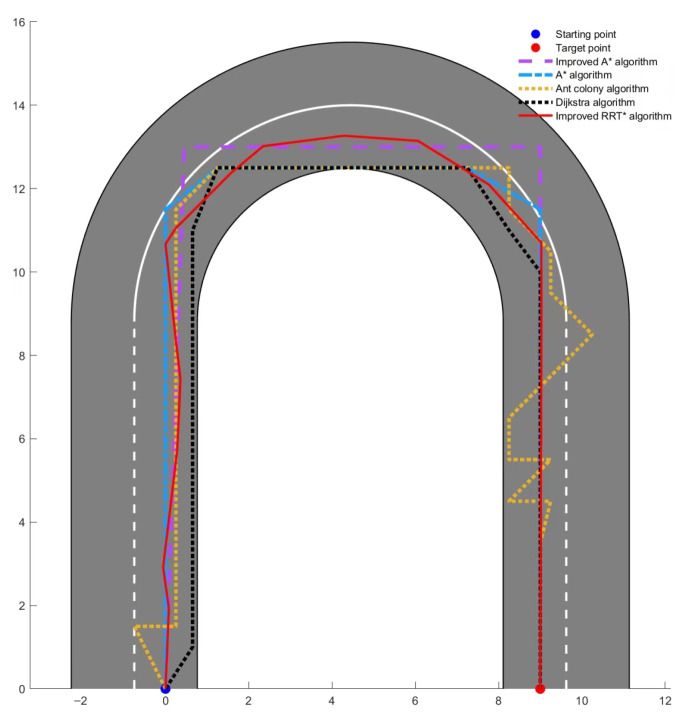
The improved algorithm in this paper and the overall effect simulation path of the four algorithms.

As depicted in Figure 22, the blue dotted line, purple dashed line, yellow dashed line, black dashed line, and red solid line are the paths planned by the A* algorithm, the improved A* algorithm, the ant colony algorithm, the Dijkstra algorithm, and the improved RRT* algorithm, respectively. it is evident that the improved RRT* algorithm can rapidly plan the global path compared to the other four algorithms while still maintaining a certain safety margin from the road boundary; it does not encroach on the opposite lane, and the vehicle tends towards the centerline of the lane in which it is situated, and the planned path has more accurate nodes, which play an accurate guiding role for the following fusion algorithm. Among them, the improved A* algorithm takes into account the lane boundary constraints. Although the Dijkstra algorithm and the A* algorithm have a slightly smaller average path length, they do not take into account the impact of the lane boundary on the path. The planned path cannot avoid driving along the lane boundary and colliding with the lane boundary, resulting in a risky path. Due to the influence of the level of pheromone, the path planned by the ant colony algorithm has some limitations, such as a return path, a long planning time, many path turning points, and so on. The enhanced A* algorithm considers the safe distance from the lane boundary and mitigates the risk of driving close to or along the boundary, but does not consider the opposite lane, resulting in the encroachment of the opposite lane; thus, the risk path appears. In comparison to the enhanced RRT* algorithm outlined in this study, the other four algorithms obviously show poor ability to deal with the turning road. The comparison of performance indicators of the five algorithms is depicted in Table 4.

#### 6.1.2. Experiment 2

To evaluate the efficacy of the enhanced RRT* algorithm’s path post-processing and the effect of the node pruning strategy and node optimization, the enhanced RRT* algorithm and the enhanced RRT* algorithm with path post-processing are both subjected to simulation experiments within identical scenarios to facilitate comparative analysis.

**Figure 23:**
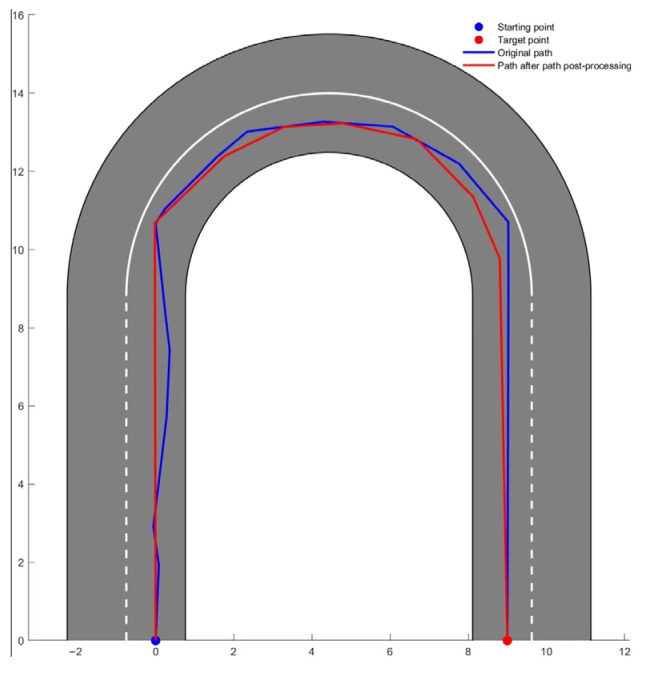
Path post-processing simulation path.

According to Figure 23, it is evident that the average number of nodes after path post-processing is 25% less than that of the original path, and the average path length exhibits a reduction of 18.32% compared to the original path. There are fewer redundant nodes in the optimized path, and the corner of the original path becomes smoother in the bend, which adheres more closely to the vehicle’s kinematic constraints, and the updated node is more accurately close towards the centerline of the lane, which lays a better foundation for the future fusion algorithm. The results indicate that the path generated by the enhanced RRT* algorithm with path post-processing is superior, which is more suitable for guiding vehicles in the future fusion algorithm and directly verifies the effectiveness of path post-processing. The comparative analysis table of path post-processing performance indicators is depicted in Table 5.

### 6.2. Simulation Analysis of Fusion Algorithm

**Figure 24:**
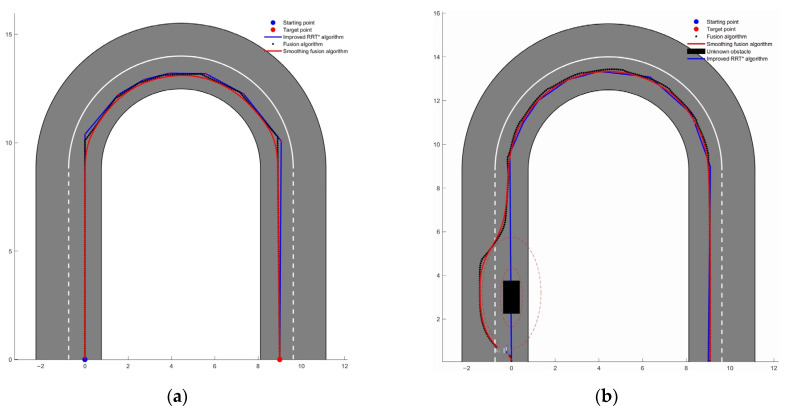

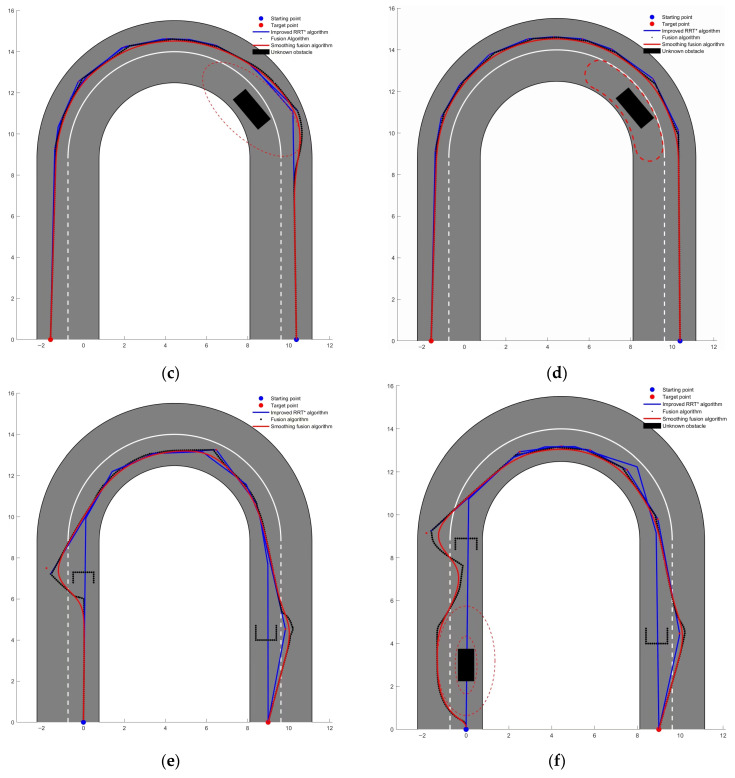
Simulation analysis of fusion algorithm: (**a**) simulation path of fusion algorithm without obstacle; (**b**) simulation path of obstacle fusion algorithm on straight road; (**c**) limited simulation path of safe ellipse of obstacles in bends; (**d**) simulation path of fusion algorithm for obstacles in bends; (**e**) simulation path of road fusion algorithm with U-shaped obstacles; (**f**) simulation path of road fusion algorithm with complex obstacles.

In order to verify the effectiveness of the improved RRT* and improved APF fusion algorithms, the road environments of turning back and S turning are established, respectively, to verify the passing ability of the fusion algorithm for turning back and S turning and to set up sudden, unknown obstacles at different positions on the road. Further analysis of the fusion algorithm ensures that it can pass through the turn while avoiding unknown obstacles smoothly. Finally, a path-smoothing strategy is introduced to the planned path. With or without unknown obstacles and unknown obstacles in straight roads or bends and road scenes with complex obstacles, the passability and obstacle avoidance ability of the fusion algorithm are analyzed and verified. Figure 24 show the simulation results of experiments under different obstacles. Among them, the blue solid line, black point, and red solid line are the paths planned by the improved RRT* algorithm, the fusion algorithm, and the smooth fusion algorithm, respectively.

Through the above five groups of simulation experiments, from Figure 24a, it is evident that when utilizing the fusion algorithm, the vehicle can navigate the curved road more smoothly than with the traditional APF algorithm. On the road without unknown obstacles, both the enhanced RRT* algorithm and the fusion algorithm can generate a path from the initial point to the final destination. However, as can be seen from Figure 24b,e, after the unknown obstacles appear on the road, the enhanced RRT* algorithm by itself cannot adequately evade abrupt unknown obstacles, while the fusion algorithm can evade the unknown obstacles and satisfy the ellipse collision constraints. As depicted in Figure 24e, when facing the first U-shaped obstacle, the vehicle is trapped in the local optimal situation. Through the action of the fusion algorithm, a virtual target point is introduced in proximity to the obstacle, and after guiding the vehicle to near the virtual target point, the global path re-planning is realized, and the temporary target point is updated to keep the vehicle going. For the second U-shaped obstacle, it is because the global path is re-planned. By making a detour, the vehicle can be guided to avoid it; additionally, the fusion algorithm demonstrates robustness in obstacle avoidance. As can be seen from Figure 24c,d, before the elliptical slot processing is introduced into the algorithm, the obstacles have some limitations in the curve, which could impact the driving of the main vehicle and make the planned path turn unnecessarily. It even makes the main car fall into the local optimal situation. After the introduction of elliptical slot processing, the path will not be disturbed by obstacles, and the vehicle can navigate the road smoothly. To further validate the efficacy of the fusion algorithm, all obstacles are put on the same turning road. As shown in Figure 24f, the fusion algorithm can avoid unknown obstacles in complex scenes and satisfy collision constraints. The two U-shaped obstacles are also reasonably avoided. When reaching near the virtual target point, it can re-plan a global path and successfully guide to the target point. Simultaneously, the path planned by the above experiment is smoothed to meet the requirements of vehicle tracking.

**Figure 25:**
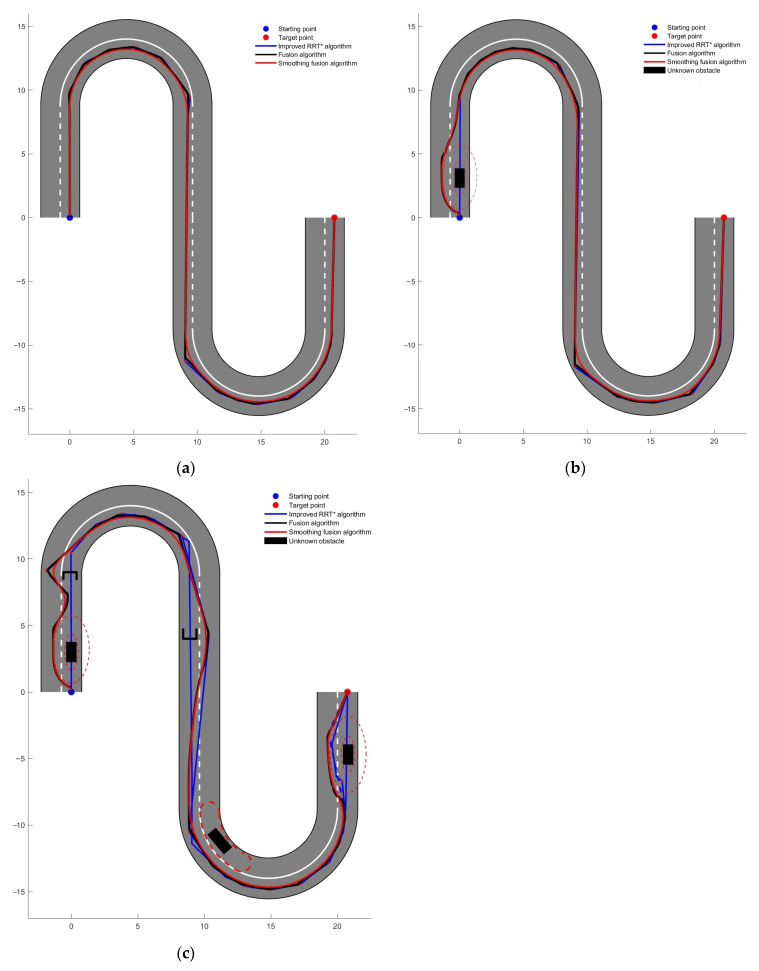
Simulation analysis of fusion algorithm: (**a**) simulation path of S-bend obstacle-free scene fusion algorithm; (**b**) simulation path of S-bend simple obstacle scene fusion algorithm; (**c**) simulation path of S-bend complex obstacle scene fusion algorithm.

To further corroborate the effectiveness and robustness of the fusion algorithm, the road scene is changed from a single U-turn to a complex S-bend. The simulation results from Figure 25 show that no matter the barrier-free S-bend road, the simple obstacle S-bend road, or the complex obstacle S-bend road, the fusion algorithm can still safely and reasonably avoid random obstacles and reach the target point. In Figure 25c, when the last obstacle is planned by the fusion algorithm, the improved RRT* is re-planned twice. In the first re-planning, the path node falls within the scope of the collision constraint, so it is eliminated. After the second re-planning, the node is outside the constraint range, and the vehicle is successfully guided to avoid the obstacle. At the same time, the above S-bend road simulation experiments ensure the smoothness of the planned path to fulfil the vehicle’s tracking requirements. All of the aforementioned experimental results underscore the indispensability and superiority of the fusion algorithm.

## 7. Conclusions

For the purpose of solving the path planning problem of highlighting unknown obstacles on the structured road, this paper takes the improved RRT* algorithm and the improved artificial potential field method as the main body of research, examines the path planning algorithm, improves the global path search efficiency and path quality of the RRT* algorithm, and solves the limitations of the artificial potential field method of local path planning. Combined with the two algorithms, a fusion algorithm is devised for global dynamic scene path planning.

(1) Targeting the deficiencies of the RRT* algorithm, such as strong randomness, slow convergence speed, poor path feasibility, and poor ability to deal with corners, the RRT* algorithm is improved, and the concepts of artificial potential field and probabilistic sampling optimization are introduced to make RRT* node sampling more purposeful, probabilistic sampling applicability stronger, and sampling efficiency better. Considering the constraint imposed by the fixed step size, the adaptive step size is designed according to the road curvature to solve the problem that oscillation may occur near the target point, improve the adaptability to each road scene, and achieve rapid convergence towards the target point accurately. The path planned by the improved RRT* is post-processed to minimize the number of redundant nodes along the path and optimize the global path quality.
(2) In view of the problems that the artificial potential field method is prone to local optimization, the target is unreachable, and it is not suitable for the global scene, an enhanced artificial potential field method is introduced, which adds obstacle avoidance constraints to obstacles and formulates a road boundary repulsion potential field to delineate the risk boundaries within the road space. The distance factor is added to the repulsion function to solve the problem of target unreachability. In the face of U-shaped obstacles, virtual gravity points are introduced to solve the local minimum problem and improve the performance of obstacle avoidance. For the case where the obstacle is located in the bend, the safety ellipse of the obstacle is treated with an elliptical slot to reduce unnecessary steering on the planned path.
(3) Aiming at the emerging unknown obstacles, complex obstacles, and other road scenes, a fusion algorithm of improved RRT* and an improved artificial potential field method is proposed. The enhanced RRT* algorithm is employed for generating the global path, and the nodes of the global path are extracted to serve as temporary target points for the artificial potential field method, which guides the vehicle to drive, reduces the occurrence of the local minimum, uses the artificial potential field method to avoid the local path when encountered with unknown obstacles, and carries on the path smoothing processing to the planned path to satisfy the vehicle’s driving. The simulation results in different scenarios demonstrate that the fusion algorithm can successfully plan a smoother and more feasible path and verify its effectiveness and robustness.

Currently, this study has its limitations. In our future work, we will consider adding the fusion algorithm to decision making in more complex road environments, such as crossroads, and analyze its path-planning effect.

## Figures and Tables

**Table 1:** Constrained range path cost.

Candidate Point	Trails	Path Distance
5	0-5-9	0-5-9 = 8
6	0-4-6-9	0-4-6-9 = 16
8	0-5-8-9	0-5-8-9 = 9

**Table 2:** Reconnection process path cost.

Neighboring Node	Original Consideration	New Pathway	New Price
4	10	0-5-9-4	0-5-9-4 = 12
6	10 + 5 = 15	0-5-9-6	0-5-9-6 = 9
8	3 + 5 + 1 = 6	0-5-9-8	0-5-9-8 = 11

**Table 3:** Comparative analysis table of performance indexes of RRT algorithm.

Algorithm	Average Path Length	Average Number of Nodes	Average Simulation Time (s)	Average Iterations
RRT algorithm	37.92	81	29.61	6150
RRT* algorithm	34.05	42	20.09	5590
Traditional improved RRT algorithm	33.89	47	2.24	575
This paper improves the algorithm.	32.47	12	1.21	279

**Table 4:** Comparative analysis table of performance indexes of five algorithms.

Algorithm	Average Path Length	Average Number of Nodes	Average Simulation Time (s)	Average Iterations
Dijkstra algorithm	31.49	7	1.42	313
A* algorithm	31.65	6	1.37	175
Ant colony algorithm	32.93	16	3.47	-
Improved A* algorithm	31.78	4	1.15	116
This paper improves the algorithm.	32.47	12	1.21	279

**Table 5:** Comparative analysis table of performance index of path post-processing.

Algorithm	Average Path Length	Average Number of Nodes
This paper improves the algorithm.	32.47	12
An improved algorithm after node optimization	26.52	9

## Data Availability

The data used to support the findings of this study are available from the corresponding author upon request.
